# A Review of Methods Used to Detect Methamphetamine from Indoor Air and Textiles in Confined Spaces

**DOI:** 10.3390/toxics10110710

**Published:** 2022-11-21

**Authors:** Gemma L. Kerry, Kirstin E. Ross, Jackie L. Wright, G. Stewart Walker

**Affiliations:** 1 Physical and Molecular Sciences, College of Science and Engineering, Flinders University, Adelaide 5042, Australia; 2 Environmental Health, College of Science and Engineering, Flinders University, Adelaide 5042, Australia; 3 Environmental Risk Sciences Pty Ltd., Carlingford Court, P.O. Box 2537, Sydney 2118, Australia

**Keywords:** methamphetamine, indoor air, textile, contamination, detection, environmental exposure, third-hand exposure

## Abstract

Methamphetamine manufacture, use, and the resulting contamination is a significant issue that affects public health, the environment, and the economy. Third-hand exposure to methamphetamine can result in adverse health risks for individuals and first responders. Such exposures can result from the inhalation of airborne residues or from contact with contaminated objects. This review was conducted to determine the current methods used for methamphetamine extraction from indoor air and porous fabric materials. Dynamic solid phase microextraction (SPME) and sorbent sampling tubes have been applied to extract airborne methamphetamine residues from contaminated properties. SPME and solvent extraction have been applied to sample clothing and textiles for methamphetamine detection. This review demonstrates that there is limited literature on the detection of methamphetamine from indoor air and clothing. Supplementary and consistent methods to detect methamphetamine from air and porous surfaces should be developed and published to allow better assessment of the environmental risk to public health caused by third-hand exposure to methamphetamine.

## 1. Introduction

**Figure 1:**
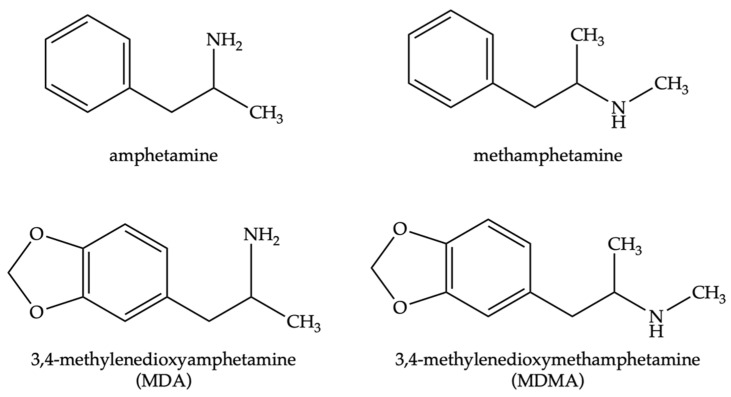
Structures of members of the amphetamine-type stimulants (ATS) class of amphetamine, methamphetamine, 3,4-methylenedioxyamphetamine (MDA), and 3,4-methylenedioxymethamphetamine (MDMA).

In 2019 1.3% of Australians aged 14 and over who responded to a survey admitted to using methamphetamine or amphetamine [1]. Overall, the United Nations Office on Drugs and Crime (UNODC) [2] reported that an estimated 0.5% of the global population aged 15 to 64 had used amphetamines. With an Australian population of 25.3 M, this extrapolates to 0.3 M Australians and 27 M individuals worldwide who are exposed to personal amphetamine use. These figures do not include those who are exposed to other peoples’ methamphetamine through second- and/or third-hand exposure. Methamphetamine is a member of the amphetamine-type stimulants (ATS) class (Figure 1) that acts on the central nervous system to release monoamines (dopamine, norepinephrine, and serotonin) [3,4]. The use of methamphetamine is related to abuse, addiction, and toxicity with adverse consequences including cardiovascular problems, paranoia, psychosis, and even mortality [3,5,6]. The drug is commonly found in four forms: a powder; pills; a sticky base; and the most potent form, crystalline shards, referred to as ice [7,8].

Methamphetamine is manufactured (referred to as “cooked”) in clandestine laboratories by a variety of methods commonly using precursors such as ephedrine, pseudoephedrine, 1-phenyl-2-propanone (P-2-P), and P-2-P precursors [8,9]. During the manufacturing process and after smoking the drug, methamphetamine residues, airborne particles, gases, and volatile organic compounds (VOCs) are released into the surrounding environment [10,11,12]. Methamphetamine can be present in the air phase as the volatile free base or absorbed into particles [13]. These residues can settle onto surfaces and are further spread by dermal, oral, or air transfer [14,15,16,17,18]. If a surface is porous or semi-porous methamphetamine may also transfer into the structure underneath and remain for days or longer periods of time [12,18].

There are three classes of methamphetamine exposure. First-hand exposure to methamphetamine users; second-hand exposure to those who do not take methamphetamine themselves but are exposed to the drug from a first-hand user; and third-hand exposure that occurs when someone lives in a house that has been contaminated either by drug use or synthesis or when in contact with a material that is contaminated. A methamphetamine-contaminated property could pose a third-hand exposure risk to individuals from skin contact, ingestion, and inhalation exposure routes. Those living or working in contaminated properties have experienced adverse health effects including respiratory irritation, eye irritation, nausea, headaches, behavioral issues, and sleep issues [19,20,21,22,23]. In Australia, guidelines for indoor surface methamphetamine residues are at 0.5 µg of methamphetamine per 100 cm^2^ and 10 µg/100 cm^2^ for residential and commercial properties, respectively [7]. These guidelines are based on dermal transfer and oral intake and do not take into consideration inhalation intake, thus these guidelines could significantly underestimate the overall environmental exposure. Wipe sampling of hard, non-porous surfaces is commonly performed to determine surface methamphetamine concentrations on contaminated items. There are a number of studies on the detection of methamphetamine from wipe sampling of indoor surfaces such as drywall, gyprock/gypsum walls, kitchen countertops, ceiling, tables, plastic, and glass [10,11,12,24,25,26].

Furthermore, exposure to contaminated clothing and textiles could be a source of third-hand exposure from dermal contact, ingestion particularly from children with hand-to-mouth behaviors, and from re-emission of methamphetamine [27]. The partitioning of methamphetamine to textile materials has been poorly studied, though studies have shown that chemicals related to third-hand tobacco smoke can sorb to clothing and re-emit [28,29,30]. Typically, soft furnishings and clothing are discarded from contaminated properties as they can be difficult to decontaminate and can re-emit residues [31,32]. However, Serrano et al. [15] demonstrated up to 99.9% methamphetamine removal after three wash cycles of contaminated clothing.

The presence of methamphetamine indoors poses environmental and public health risks [21,22,23,33,34,35]. While there are studies that describe a variety of methods for sampling and detecting this drug in biological or environmental matrices, illicit samples, or on contaminated surfaces [36,37,38,39], there is less in the literature on methamphetamine extraction and detection from indoor air and porous samples such as fabrics. To aid in the understanding of transfer and possible exposure routes from indoor air and fabrics, this review reports on current methods for extraction and detection of methamphetamine from indoor air and textiles. This review serves to highlight that standardized protocols for extracting and detecting methamphetamine from indoor air and porous samples such as fabrics are needed.

## 2. Materials and Methods

Four databases Scopus, Web of Science, PubMed, and Google Scholar were searched for articles containing keywords including variations of methamphetamine (Table 1) written in English and published prior to November 2022. Articles were initially screened using Covidence systematic review software (Veritas Health Innovation, Melbourne, Australia) by reading titles and abstracts and excluding papers if they were review articles, theoretical work, focused on biological matrices or wastewater, or if they did not specifically refer to methamphetamine. If it was unclear whether articles met inclusion criteria they were included for full-text review. Articles were then read in full and included in the study if they described methamphetamine extraction, detection, or quantification from indoor air, or methamphetamine extraction, detection, or quantification from clothing/textiles. Articles were excluded if they did not focus on methamphetamine or were a different sample type or used biosensors. Articles were also excluded from air studies if they were controlled laboratory experiments. Subsequent snowball searching using articles’ references was performed to identify studies focusing on indoor air or textile sampling for methamphetamine extraction and detection.

All papers included in the study had key points extracted and recorded along with the country where the sample was taken, the extraction and detection methods used and the sample type.

## 3. Results

**Figure 2:**
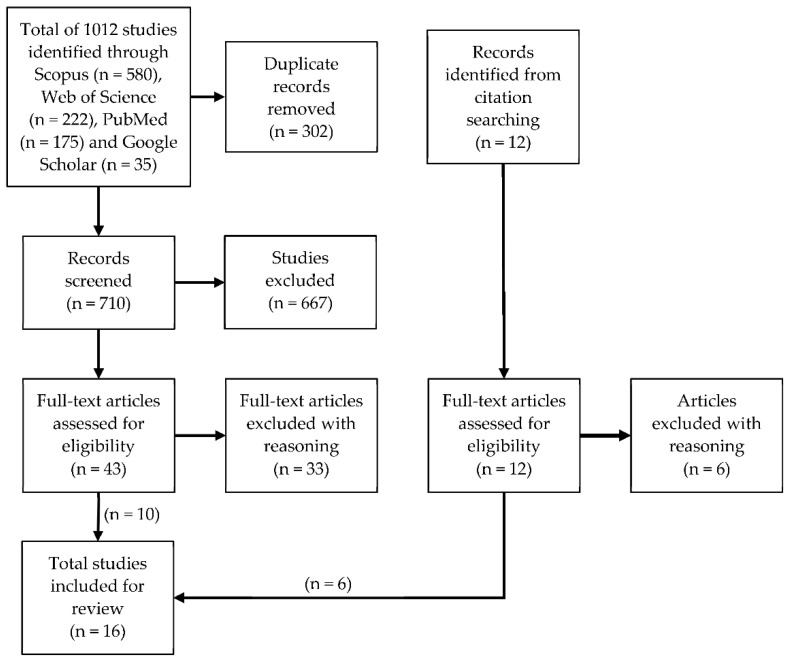
Literature search flow diagram presenting the exclusion process for studies showing the total number of articles (n = 16) identified as eligible.

Using the search terms 1012 papers were retrieved from Scopus, Web of Science, PubMed, and Google Scholar databases with ten studies identified as eligible for inclusion. A further six articles were included based on subsequent snowball searching of references (Figure 2). Of the 16 studies included for review, eight studies were identified as sampling indoor air (Table 2) while 11 studies were identified as sampling clothing or textiles (Table 3). Of the 16 studies, three studies sampled both indoor air and textiles. Thus, this systematic review has shown that the research on both indoor airborne methamphetamine extraction and extraction of methamphetamine from textiles has been limited. It was noted that a large number of papers were published that relate to sampling, extraction and detection of methamphetamine on indoor hard surfaces [10,11,12,24,25,26]. While these are not directly relevant to this review, there are some commonalities in extraction methods to the papers evaluated.

## 4. Discussion

### 4.1. Extraction and Detection of Indoor Airborne Methamphetamine from Manufacture

Sampling for airborne particulate matter, VOCs, and aerosols is commonly performed using active air sampling with sorbent tubes. Typically, active sampling involves drawing a known air volume through a sorbent in a glass tube using a pump to trap the analyte onto the sorbent. Thermal or solvent desorption is then performed followed by instrument analysis. A variety of solvents can be used for analyte desorption [48].

Air-phase methamphetamine can be present as airborne methamphetamine particles or as the more volatile free base, leading to inhalation exposure. Currently, no guidelines include recommendations for sampling and analyzing indoor airborne methamphetamine [7,49].

Non-peer-reviewed, internal studies have been conducted by the National Jewish Medical and Research Center for methamphetamine detection in air [50,51,52,53,54]. These studies were summarised and published in peer-reviewed papers by Martyny et al. [11,12]. Martyny et al. [12] sampled air in a property after controlled methamphetamine manufacture using red phosphorus, hypophosphorous, phosphorus flake, and anhydrous ammonia synthesis methods. Sampling was performed using a sampling pump set at 2 L/min flow rate with acid-treated glass fiber filters near where the cook took place and at a distant sampling area. Samples were then desorbed and analyzed by gas chromatography-mass spectrometry (GC-MS). The authors reported detecting airborne methamphetamine at levels from 79 µg/m^3^ to 5500 µg/m^3^ in the cook area and levels at or below 4200 µg/m^3^ at distant sampling locations. Van Dyke et al. [16] performed a similar study by extracting airborne methamphetamine onto three different media during and after methamphetamine manufacture using a red phosphorus manufacture method. Total airborne methamphetamine levels were reported at 520 and 760 µg/m^3^ in the cooking area collected after two red phosphorus methods using the same air sampling method and filters as Martyny et al. [12]. These results were lower compared to a previous average of 1524 μg/m^3^ airborne methamphetamine from controlled manufacture [12] possibly indicating that the methamphetamine levels from a typical clandestine laboratory were underrepresented in this study.

Van Dyke et al. [16] also reported most airborne methamphetamine was of a respirable size with concentrations at 720 and 780 µg/m^3^ based on respirable air sampling and size distribution air sampling for two red phosphorus cooks. Both Martyny et al. [12] and Van Dyke et al. [16] suggested that methamphetamine was released during the “salting-out” process of methamphetamine synthesis based on methamphetamine surface distribution and airborne size distribution.

While these studies attempted to mimic methamphetamine clandestine laboratory manufacture, not all indoor factors can be controlled. Environmental conditions such as temperature and humidity along with indoor conditions such as air quality, ventilation, heating, and air conditioning could affect methamphetamine desorption and transfer. For example, a laboratory study reported an increase in methamphetamine transfer from gyprock/gypsum wall materials into the air phase with increased temperature and increased relative humidity [55]. Since methamphetamine concentrations would be expected to vary depending on factors such as clandestine laboratory activity and manufacturing methods, air quality, ventilation, and environmental factors, further comparisons between indoor airborne methamphetamine levels and known manufacturing methods are needed.

While the studies by Martyny et al. [12] and Van Dyke et al. [16] of controlled methamphetamine manufacture are important for the representation of airborne methamphetamine levels, they are not necessarily representative of methamphetamine release under conditions from former clandestine laboratories. Consequently, Wright et al. [13] tested for airborne methamphetamine in a previously contaminated property from suspected methamphetamine manufacture [18,22]. Personal sampling pumps at a 1 L/min flow rate connected to ORBO^TM^-49P treated Amberlite^®^ XAD^®^-2 resin sorbent tubes were used for sampling of the property. The sorbent tubes were analyzed based on the National Institute for Occupational Safety and Health (NIOSH, United States) method 9111 which is used for the analysis of wipe samples [56]. The sorbent tubes used are designed for sampling organophosphorus pesticides in vapor and aerosol form [57] but were chosen in this study because methamphetamine and methamphetamine hydrochloride are semi-volatile organic compounds. Air phase methamphetamine concentrations for the property were between 0.53 to 8.3 µg/m^3^ when sampling in different rooms and sampling with the air conditioner turned on or off. Differences in sampling volume of 132 to 286 L due to limited time access in the property could account for the concentration range. Nevertheless, methamphetamine recovered from the sorbent tubes after air sampling of this house suggested that total methamphetamine intakes by young children and adults could be 0.01 mg/kg/day and 0.001 mg/kg/day respectively, from oral, dermal, and inhalation exposures, with intakes from inhalation comprising 20% for young children or 80% for adults of total intake [13]. Intake values were calculated using approaches adopted for calculating methamphetamine intake via dermal absorption and methamphetamine residue ingestion from hands and objects (for children), along with potential intake from inhalation [49,58]. Total exposure values will vary based on individuals and these values are only applicable to the property sampled.

Interestingly, in an internal report by Raynor and Carmody [40] a sampling train was developed to analyze methamphetamine from contaminated properties. The sampling train consisted of a sampling pump set at 1.5 L/min flow rate connected to a silica gel sorbent tube followed by a glass fiber filter in a styrene cassette. Samples were desorbed using methanol for analysis by liquid chromatography-mass spectrometry (LC-MS). No airborne methamphetamine was detected at one former methamphetamine laboratory that was occupied for three years and partially renovated, however detectable amounts of the drug were present from wipe samples of heating, ventilating, and air conditioning (HVAC) systems. This suggested that the methamphetamine from the HVAC system was not readily vapourised or aerosolized, though sampling at times when the system is on could yield a different result. Methamphetamine was detected at low levels on glass filters and on one of four sorbent tubes at a second former clandestine laboratory several months after drug manufacture. Based on assumptions in breathing rate and exposure time the authors determined that the potential inhalation dose from this site would be 15 µg/day of methamphetamine.

While active air sampling using sorbent tubes and filters was found to provide reliable results, an assessment of gas phase methamphetamine recovery was not included in these studies. Sorbent media used in active air sampling of airborne methamphetamine were acid-treated glass fiber filters, XAD^®^-2 resin, and silica gel. The use of different media highlights the need to examine more selective and sensitive sorption media for airborne methamphetamine. The choice of a suitable sorbent depends on the physical and chemical characteristics of the analyte and on the sampling conditions such as sampling time and sample volume. Additionally, the choice of solvent used for desorption can be important for optimum recovery [59], though little to no work has been conducted to compare methamphetamine desorption with different solvents and sorbent types. Sulfuric acid was commonly used as the desorption solvent [11,13,16] in accordance with NIOSH methods, though methanol has also been used [40]. Some commonly used sorbent tubes are affected by humidity, and breakthrough volume for porous polymers can vary with temperature [59]. Further work should look at different adsorbents coupled with extraction solvents that may be more selective and sensitive for methamphetamine trapping and desorption.

Since sorbent tubes used with solvent desorption are not reusable [59], SPME has been developed as an alternative method for sampling VOC traces in indoor air [48,60,61]. The SPME technique was initially developed [62] for effective sample collection, extraction, and introduction without the use of solvents [63,64]. For fiber SPME the analyte of interest partitions into the SPME fiber coated with an appropriate stationary phase. The analytes can then be thermally desorbed into an analytical instrument, commonly a gas chromatograph [63]. SPME has been used to detect methamphetamine from headspace sampling [61,65,66] but there are limited applications for airborne methamphetamine sampling.

Preliminary work by McKenzie et al. [42] determined that SPME could be used as an air sampling method. For this, the authors performed passive and dynamic SPME sampling in a custom-made glass chamber coupled to a methamphetamine vapor generation unit comprising a mass flow controller, a vaporization unit, and a syringe pump. Time dependence methamphetamine absorption testing determined that a 100 µm poly(dimethylsiloxane) (PDMS) coated fiber showed little analyte loss when sampling for under two hours. However, the same authors also determined that adsorption differences can occur between SPME PDMS fibers from carry-over.

Brown et al. [67] have used on-sorbent derivatization as a means to concentrate volatilized methamphetamine exposed to an SPME fiber and convert the drug to a form amendable to separation using GC. The authors were reportedly able to partially resolve the two isomers of methamphetamine of S(+) and R(−) to provide information about potential starting materials (since precursor P-2-P produces a racemic mixture whereas precursor pseudoephedrine, where the chiral center is preserved, produces only one isomer).

In comparison, Gura et al. [68] implemented a dynamic planar SPME (PSPME) device with a direct introduction into an ion mobility spectrometry (IMS) instrument for the detection of piperonal, a common starting material in 3,4-methylenedioxymethamphetamine (MDMA) synthesis. MDMA tablets were placed in quart cans and the headspace was sampled using the PSPME device. Overall, when compared with SPME fibers under the same conditions the PSPME device was found to have higher extraction efficiencies for the piperonal monomer and dimer product ions.

To detect volatilized methamphetamine from contaminated properties McKenzie et al. [41] described a dynamic SPME sampling method coupled to GC-MS. The dynamic SPME sampling device was made in-house by connecting an SPME fiber holder to stainless steel tubing and coupling it to a personal air sampling pump set at 1 L/min flow rate. Air sampling was performed for a restricted sampling time of 5–30 min at 11 suspected clandestine laboratories using the dynamic sampler operated at experimenter chest level in what was described by the authors as a typical intake zone for breathing. Surface wipe samples were also taken from nine of the 11 suspected clandestine laboratories to enable comparison between surface and air concentrations. Airborne methamphetamine could be detected when analyzing extracted ion chromatograms with the main ion fragment of 58 amu at sites where surface contamination exceeded 40 μg/100 cm^2^. However, the short-term sampling was not sensitive enough to detect methamphetamine in the air when surface concentrations were between 0.05 to 1.5 μg/100 cm^2^ of the recommended guideline limits for surface contamination in different countries and states [7,69,70]. Methamphetamine detected using the dynamic air sampler suggested that methamphetamine air exposure can arise from a contaminated building. The authors, however, did not quantify the drug.

A laboratory study by Nair and Miskelly [71] later determined that a method called capillary microextraction (CME) of volatiles (CMV) used in the analysis of drugs [72], explosives [73,74] and VOCs [75] would allow for airborne methamphetamine sampling. Nair and Miskelly [71] used a CME device consisting of PDMS-coated glass filter strips inside a glass tube to dynamically sample methamphetamine vapor produced in a custom-built vapor dosing system. Rapid sampling and increased sensitivity of 30 times were reported by the authors as compared to the dynamic SPME method coupled with GC-MS analysis under identical sampling conditions [41,42]. The same authors later [76] reported on-sorbent derivatization of methamphetamine on the CME device for improvement in chromatographic peak shape and additional improvement in sensitivity. This device, however, has not yet been used for sampling in the field for determining airborne methamphetamine concentrations from suspected contaminated premises.

Overall, the indoor air studies showed that surface wipe sampling can underestimate the methamphetamine exposure risk for individuals in contaminated properties. Surface wipe sampling typically gives a large range of <0.1 to 16,000 μg/100 cm^2^ of methamphetamine depending on manufacture method, surface type, and location [12,15], while airborne methamphetamine concentrations ranged from 0.53 to 5500 µg/m^3^ depending on the manufacture method and sampling location [12,13,16]. While only two studies calculated concentrations of methamphetamine that an individual could be exposed to the respective contaminated properties sampled [13,40], this presence of methamphetamine could lead to third-hand exposure and associated adverse health effects [19,20,22,23]. Further studies on inhalation exposure to airborne methamphetamine and associated health risks from contaminated properties, particularly contaminated from former clandestine laboratory manufacture, would be beneficial. Such studies could investigate the impact of the methamphetamine particle size on the respiratory process (such as possible differences in adsorption in the nostril, throat, lungs, and stomach). In addition, further research on the forms of methamphetamine in the air as either the vapor or particulate form would be beneficial.

### 4.2. Extraction and Detection of Indoor Airborne Methamphetamine from Smoking

Methamphetamine manufacture and methamphetamine smoking release different residues and VOCs into the air. While the previously mentioned studies focused on air sampling in properties with suspected, known, or controlled clandestine methamphetamine manufacture, Martyny et al. [11] studied the release and detection of airborne methamphetamine after simulated “smoking” of the drug. The authors found that airborne levels ranged from 300 to 520 μg/m^3^ with a single smoke of 100 mg of methamphetamine (91% purity) when using the same air sampling procedure as previously mentioned [12]. These values however would be expected to be lower if a smoker were present to absorb the methamphetamine. Additionally, an increase in the number of smokes or the total amount smoked would increase the levels of airborne methamphetamine.

A recent study by Russell et al. [77] examined contamination levels on building surfaces after simulated smoking events of in-house-produced methamphetamine hydrochloride. Surface values of 0.25 to 2.96 µg/cm^2^ were obtained after simulated smoking of 0.2 g methamphetamine, with varying concentrations obtained from the different materials surface wipe sampled. In addition, it was found that methamphetamine levels decreased significantly after a four-week period from the final smoking event.

Given the above, it is likely that contamination levels from methamphetamine smoking would be lower compared to that of methamphetamine manufacture. It is necessary, however, to provide more information on airborne methamphetamine contamination levels from smoking as compared to methamphetamine manufacture to characterize risk and provide suitable guidelines for overall methamphetamine contamination levels in a property.

### 4.3. Clothing and Textile Methamphetamine Extraction and Detection

Exposure to contaminated clothing and textiles can be a source of third-hand exposure, so it is beneficial to understand the transfer and emission of methamphetamine to textiles. Typically, solvent extraction or SPME followed by GC-MS or LC-MS analysis is used for compound analysis on fabrics, though there are no universally recommended guidelines for extracting methamphetamine from textile materials.

Methamphetamine can be excreted in sweat, so Al-Dirbashi et al. [44] extracted the drug from abusers’ clothing using solvent extraction. Analysis was performed using high-pressure liquid chromatography (HPLC) with ultraviolet (UV) or fluorescence (FL) detection. Methamphetamine was detected in underpants, undershirts, and pant samples belonging to known users. Higher concentrations from the undershirt and underpants compared to the pants was referred to the direct skin and skin secretion contact. Similarly, Keasey [46] detected methamphetamine residues from a range of clothing samples submitted from suspected and known users after liquid extraction and GC-MS analysis. Liquid extraction methods can be laborious therefore, Talaty et al. [45] used desorption electrospray ionisation (DESI) coupled to MS for direct and rapid analysis of explosive and drug compounds on textiles. A drug mixture of 2.5 ng each of methamphetamine, cocaine, and heroin was spotted on 100% cotton (natural fiber) and 100% polyester (synthetic fiber) samples. While methamphetamine was detected from both cotton and polyester samples based on the presence of the protonated molecular ion [M + H]^+^ at *m*/*z* 150, quantification was not performed. These studies did not investigate the recovery and sorption of the different clothing materials.

Quantification of methamphetamine extracted from clothing was performed by Martyny et al. [12] after controlled methamphetamine manufacture using phosphorus and anhydrous ammonia methods. Surface wipe sampling of the front and back sections of protective clothing worn by cook participants showed methamphetamine ranging from 0.2 μg/sample to 150 μg/sample. It was proposed that wipe concentrations from the back areas were likely due to airborne methamphetamine rather than splatter during manufacturing. This suggested that airborne methamphetamine released during manufacture can transfer and contaminate clothing.

Splatter or spillage of organic solvent and methamphetamine could also account for methamphetamine contamination on clothing. One study stated that a majority of the surface contamination from methamphetamine manufacture using a “one-pot” method could be accounted for by spillage of organic solvent during and post-cook [24]. This study by Ciesielski et al. [24] investigated surface contamination generated from one-pot methamphetamine manufacture where an approximate 100 cm^2^ area of personal protective equipment (PPE) worn by researchers was sampled for analysis by lateral flow immunoassay and fluorescence covalent microbead immunosorbent assay. The authors concluded that swab samples had the highest post-cook methamphetamine concentrations from the tabletop where the manufacture was conducted and from the PPE of the researchers performing the manufacture, particularly from legs and belt areas which were suggestive of splatter or leaning against the contaminated tabletop.

Serrano et al. [15] investigated the effect of cleaning contaminated clothing. Clothing was contaminated with aerosolized street-manufactured methamphetamine then sample swatches were liquid extracted and analysed by LC-MS. Mean pre-wash methamphetamine concentrations were 205 μg/100 cm^2^ and 120 μg/100 cm^2^ for loose-weave cotton and tighter-weave denim, respectively. Nomex^®^ coveralls had a concentration range of 160 to 570 μg/100 cm^2^ while polyester/cotton overalls had a range of 83 to 880 μg/100 cm^2^ of drug. These findings showed that further comparison on sorption between differing materials is needed, as methamphetamine concentrations on fabrics can vary. Interestingly, Serrano et al. [15] found that washing clothing with commercial detergent and a household washing machine effectively removed greater than 90% of methamphetamine, comparable to findings stated by Al-Dirbashi et al. [44]. This could mean that methamphetamine is not irreversible bound to fabric fibers, thus, the drug could re-emit.

Calculating partition coefficients between fabric sorbent and analyte in the air is a common method to investigate sorption behavior. Morrison et al. [27] conducted a study to detect the sorption of methamphetamine from the air phase on clothing and toy fabrics. The method consisted of exposing fabric samples to a stream of gaseous methamphetamine in a sealed chamber and then liquid extracting the fabric samples. Partition coefficients at 30% relative humidity were obtained of 29, 18, and 5.5 µg/(g ppb) (units of µg of methamphetamine per gram of substrate per ppb) for cotton, cotton/polyester blends, and polyester fabrics, respectively. Results were similar to those from Noble [29] who found cotton accumulated up to 10 times more tobacco smoke by sample mass compared to polyester. Relatively large partition coefficients showed that low air concentrations of methamphetamine could accumulate on fabrics and exceed recommended methamphetamine concentrations for surfaces. The absorbency of a fabric depends on chemical and physical properties [29], so cotton with more polar sites more strongly absorbed polar methamphetamine than polyester comprising fewer polar sites [78,79]. Interestingly, Morrison et al. [27] did not detect a measurable increase in partition coefficients for skin oil-soiled cotton, though skin oil spread on a polytetrafluoroethylene (PTFE) filter increased the sorptive capacity of the filter. This was hypothesized to arise from a difference in partition coefficient for different skin oils obtained, or that adsorption to fiber sites was greater than absorption into a surface coating. Furthermore, another study found that loose-weave cotton material appeared to readily desorb methamphetamine without treatment [15], indicating that methamphetamine sorption and desorption from different fabric materials are not well investigated. The NIOSH method 9106 for surface wipe sampling to detect methamphetamine recommends using a cotton gauze, as cotton was found to be good or better than five synthetic fabric gauzes for the wipe media [80,81].

Sorption and desorption of methamphetamine and a methamphetamine surrogate from wallboard/drywall have been studied. Li [55] determined that the methamphetamine to gypsum equilibrium partition coefficient ranged from 1.1 to 3.0 × 10^−05^ (µg methamphetamine/m^3^ gypsum)/(µg methamphetamine/m^3^ air) for drywall with temperatures of 20 to 30 °C and relative humidity of 19% to 68%. A range in the partition coefficient is representative of the differences in conditions and drywall materials. The same author [55] also reported an increase in airborne methamphetamine transfer from drywall materials with increased temperature and increased relative humidity. Furthermore, Poppendieck et al. [14] used a methamphetamine surrogate, n-isopropylbenzylamine (NIBA) to study desorption from wallboard. While this approach was not intended to simulate contamination from clandestine laboratories, emission rates were reported at 35 to 1400 µg h^−1^ m^−2^ after 15 days from contamination. The authors concluded that elevated temperatures and encapsulation of the wallboard with latex paint will not significantly reduce methamphetamine emissions from the wallboard. These studies showed that methamphetamine can re-emit from building materials.

Salocks et al. [47] investigated the transfer of carbon-14 labeled methamphetamine hydrochloride from contaminated vinyl tile and fabric to human skin in vitro. For this, a spiked fabric disk was placed in contact with a skin surface in a diffusion cell and then measured for radioactivity using a liquid scintillation spectrometer. While this study did not investigate sorption capacity into the fabric, this study determined the radioactivity recovered as a percent applied dose during skin and fabric disk contact time. Five minutes after skin and fabric disk contact the [^14^C]-methamphetamine hydrochloride was observed in skin and receptor fluid samples, indicating that fabric-to-skin drug transfer was rapid. In addition, the moistened fabric was found to have seven times greater fabric-skin transfer efficiency compared to the dry fabric after two hours of contact.

Fabric materials with a large surface area such as carpeting fabrics can act as a sink and contaminant source [82]. Vacuum samples of carpet prior to methamphetamine manufacture and approximately 13 h after by Van Dyke et al. [16] resulted in methamphetamine levels between 2.7 to 5.5 µg/m^2^ and 54 to 270 µg/m^2^, respectively. Post-cook results showed greater levels of methamphetamine in the cooking area and highlighted that methamphetamine can transfer to surfaces between rooms. Subsequent research by Van Dyke et al. [17] found surface wipe sampling and subsequent bulk analysis of carpet sections gave concentrations in the range of 5.4 to 70 μg/100 cm^2^ and 27 to 460 μg/100 cm^2^, respectively. In comparison to lower residual contamination from linoleum and drywall, the carpet appeared to readily absorb methamphetamine resulting in higher surface contamination. Similarly, after bulk analysis, Wright et al. [18] concluded that methamphetamine concentrations were significantly elevated in carpet materials from a suspected former clandestine laboratory. These findings indicated that the methamphetamine in the property was highly transferable and persistent.

Additionally, Wright et al. [13,18] compared bulk analysis and air sampling of soft toys obtained from a contaminated property. Methamphetamine concentrations ranged from 1.1 to 12 μg/g for bulk analysis and 0.046 to 0.3 µg/m^3^ for air sampling of the toys in a sealed bag. Both liquid extraction and air sampling of soft toys showed that methamphetamine could sorb and desorb from these toy materials.

In a study by Bitter [10] silicon, plastic, laminate, and artificial leather surfaces were extracted in methanol after contamination with volatilized methamphetamine. The porous artificial leather had extracted methamphetamine recovery significantly lower compared to the other substrates. The author concluded that a better extraction method particularly for the porous artificial leather may yield better recoveries.

The textile articles in this review used different solvent extraction solutions commonly containing sulfuric acid, chloroform, methanol, and water. In the NIOSH methods 9106 and 9111, desorption of methamphetamine and other drugs from wipe sample media occurs with 0.1 M sulfuric acid. Al-Dirbashi et al. [44] compared different solvent chloroform:propan-2-ol combinations of 2:1, 3:1, 5:1, and 9:1 (*v*/*v*), along with methanol:5 M hydrochloric acid (HCl) (20:1 *v*/*v*) and 1 M HCl solvents to optimize methamphetamine extraction from clothing. A mixture of chloroform:propan-2-ol in a 3:1 *v/v* ratio was chosen for extraction due to the high relative drug recovery and shorter solvent evaporation time. Similarly, Keasey [46] compared two extraction methods. The author extracted methamphetamine and other drugs from non-probative clothing samples with one method using water, HCl, and chloroform solvents, and the second method with HCl, sodium hydroxide (NaOH), and chloroform solvents with shaking overnight. As reported by the author, the first method did not allow for the detection of methamphetamine from non-probative samples, however, the second method allowed more time for the low levels of drugs to diffuse from the fabrics into the solvent [46]. The increased incubation time of ≥9 h of a fabric sample in the extraction solvent was found to increase drug recovery in the work of Al-Dirbashi et al. [44]. While an extensive comparison between solvents for desorption from textiles has not been undertaken in the literature, preliminary work suggests that an increased time in the extraction solvent could increase drug recovery. As different organic and aqueous solvents have been used in the extraction of methamphetamine from fabrics, further study is needed to form a guided method on solvent choice and extraction time.

Overall, sizing, filler agents, and chemical additives as well as physical properties of textiles could contribute to differences in methamphetamine sorption and desorption. In addition, relative humidity influences sorption and desorption depending on the material surface [83,84]. Methamphetamine sorption and desorption between different chemical and physical properties of textiles should be investigated further. Knowing the sorption and desorption of methamphetamine from textiles will provide a better understanding of the third-hand exposure impacts on individuals. Furthermore, effective remediation of clothing should be examined further.

### 4.4. Detection Methods

There have been many publications relating to the use of a variety of chromatographic and mass spectrometric techniques for the detection, identification, and quantification of amphetamine-type stimulants [13,44,45,56,85,86]. Al-Dirbashi [44] used high-performance liquid chromatography (HPLC), whereas gas chromatography or liquid chromatography were commonly used by articles in this review [11,12,13,16,41]. NIOSH methods 9106 and 9111 [56,80] were methods used in references [11,13,15,16,17,18] that used GC or LC techniques, respectively. These NIOSH methods have examples of instrument methods that can be used for the analysis of methamphetamine and illicit drugs on surfaces.

Sample preparation before chromatographic analysis can include clean-up and/or derivatization. While GC is a widely accepted method for separation, ATS are derivatized to make the compounds less polar and more volatile which helps improve the stability, separation, and peak shape [87,88,89]. Common derivatizing agents used for ATS include pentafluoropropionic anhydride (PFPA), heptafluorobutyric anhydride (HFBA), and trifluoroacetic anhydride (TFFA) [90,91]. For methamphetamine, carbamate derivatives are favorable as the derivatives are formed at room temperature and are relatively stable [76]. LC, on the other hand, does not require derivatization [56,92,93], so can be a more efficient technique in this regard compared to GC.

## 5. Conclusions

While methamphetamine has been widely extracted and analyzed from surfaces and biological matrices, there is a lack of information in the literature concerning extraction and detection from indoor air. From air sampling studies, elevated airborne methamphetamine levels were present in indoor air at suspected former clandestine laboratories, after controlled methamphetamine manufacture, and after controlled smoking. Extraction of methamphetamine from the air has been performed using glass fiber filters and XAD^®^-2 resin and silica gel sorbent tubes connected to personal sampling pumps and dynamic SPME. However, little to no work has been performed to compare sorbent or air-trapping media, or desorption solvents. Overall, air sampling studies indicated the transfer of methamphetamine could lead to third-hand exposure from inhalation, adding to dermal and ingestion exposure routes. Currently, surface wipe sampling is used to provide information on the extent of contamination in residential and commercial properties, with guidelines set for recommended levels. These guidelines, however, do not consider airborne methamphetamine levels. Further research on comparing airborne methamphetamine levels from different manufacturing and smoking methods is needed to better understand inhalation exposure.

Similarly, there is a lack of information on methamphetamine sorption and desorption from a range of clothing and fabric materials. These processes would be important to consider as they could contribute to an individuals’ methamphetamine third-hand exposure through re-emission, direct skin contact, and potential ingestion from hand-to-mouth activity. Extraction of methamphetamine from textiles is commonly performed using solvent extraction, though further study in optimizing solvent extraction is needed to form a better representation of drug levels on varying samples. By better understanding sorption and desorption from a variety of fabrics, the risk to public health can be minimized.

The scarcity of publications outlined in this review highlights that more research needs to be completed and disseminated so that the total impact of second- and third-hand exposure to methamphetamine is known by police, forensic investigators, first responders, and legal and medical practitioners.

## Figures and Tables

**Table 1:** Search terms and keywords used to identify relevant literature from databases Scopus, Web of Science, PubMed, and Google Scholar.

Search Terms	
Key Words	amphetamine OR amfetamine OR methamphetamine OR metamfetamine OR methylamphetamine OR “clandestine laborator *” air OR “indoor air” OR airborne OR vapo?r OR “vapo?r phase” OR “air phase” OR “gas phase” OR gaseous OR volatile OR volatili?ation OR “free base” OR “meth * smok *” OR cloth * OR fabric OR textile OR upholstery
AND title/abstract/keywords	

‘*’ and ‘?’ indicates a wildcard symbol used when variations of a search term were possible.

**Table 2:** Summary of indoor air studies identifying the sample type, indoor air extraction methods, desorption methods, and detection methods.

Sample Type	Air Extraction Method	Media Desorption	Detection Method	State, Country	Reference
Two former clandestine laboratories.	Personal sampling pump (1.5 L/min flow rate) connected to a silica gel sorbent tube followed by glass fiber filter in a clear styrene cassette.	15 mL methanol	LC-MS Agilent 1100 LC-MSD	Minnesota, USA	Raynor and Carmody 2006 [40]
Suspected clandestine laboratories and controlled methamphetamine manufacture in abandoned structures using red phosphorus, hypophosphorous, phosphorus flake, and anhydrous ammonia methods.	Personal sampling pump (2 L/min flow rate) with 37 mm sampling cassettes and acid-treated glass fiber filters.	Method under development at time of paper publication.	GC-MS Make not specified	Colorado, USA	Martyny et al. 2007 [12]
Controlled “smoking” of methamphetamine in a dwelling.	Personal sampling pump (2 L/min flow rate) with acid-treated glass fiber filters.	As specified in NIOSH draft method 9106, a method used for methamphetamine analysis on wipes by liquid-liquid extraction.	GC-MS Make not specified	Colorado, USA	Martyny et al. 2008 [11]
Controlled methamphetamine manufacture in a home using a red phosphorus method.	Personal sampling pump (2 or 2.5 L/min flow rate) with closed-face, acid-treated glass fiber filter cassette and with an aluminum cyclone filter cassette. Sioutas Personal Cascade Impactor (9 L/min flow) with acid-treated glass fiber media.	As specified in NIOSH draft method 9106.	GC-MS Make not specified	Colorado, USA	Van Dyke et al. 2008 [16]
Suspected former clandestine laboratories.	Dynamic SPME field sampler coupled to an air sampling pump (sampling for 5–30 min at 1 L/min flow rate).	SPME fiber introduced into GC inlet set at 250 °C.	GC-MS HP 6890 GC + HP 5973 MS Positive Ion Mode, 70 eV	New Zealand	McKenzie, Miskelly and Butler 2013 [41,42], McKenzie 2014 [43] *
Contaminated home from known methamphetamine manufacture.	Personal sampling pump (1 L/min flow rate) with ORBO™-49P (XAD^®^-2 resin) sampling tubes.	Based on NIOSH method 9111, a method used for methamphetamine analysis on wipe samples.	LC-MS Agilent 1290 Infinity UHPLC system 6460/6470 Triple Quadrupole, LC-MS electrospray source	Australia	Wright et al. 2021 [13]

Note: GC-MS = gas chromatography-mass spectrometry, LC-MS = liquid chromatography-mass spectrometry, NIOSH = National Institute for Occupational Safety and Health, SPME = solid phase microextraction, UHPLC = ultra-high performance liquid chromatography. * Three papers were identified but merged in this summary table as they were conducted by the same authors following similar methods.

**Table 3:** Summary of textile and clothing studies identifying the sample type, sample composition and contamination, and the extraction and detection methods.

Sample Type	Sample Composition and Contamination	Extraction Method	Detection Method	State, Country	Reference
Clothing	100% cotton garment as control spiked with 0.8–1492.4 ng methamphetamine/0.1 g sample. Methamphetamine users’ clothing.	Liquid extraction of swatches using 1 mL chloroform:propan-2-ol (3:1, *v*/*v*).	HPLC-FL system Shimadzu LC-10AD*vp* pump with Shimadzu RF_10AXL spectrofluorometer HPLC-UV system Tosoh CCPD pump with Waters 484 absorbance detector	Japan	Al-Dirbashi et al. 2001 [44]
Clothing	Protective clothing worn during controlled methamphetamine manufacture in a property using red phosphorus, hypophosphorous, phosphorus flake, and anhydrous ammonia methods.	Method under development at the laboratory at time of the paper publication.	GC-MS Make not specified	Colorado, USA	Martyny et al. 2007 [12]
Textile	100% cotton and 100% polyester samples spiked with 2.5 ng each of cocaine, heroin, and methamphetamine.	Not applicable.	DESI-MS Prosolia Omni Spray™ ion source and Thermo Electron LTQ MS	Indiana, USA	Talaty et al. 2008 [45]
Textile	Carpet in a property after controlled methamphetamine manufacture using a red phosphorus method.	Carpet vacuumed using a Eureka Sanitare Commercial vacuum cleaner fitted with a Mitest Dust collection device. Samples then underwent extraction according to NIOSH draft method 9106, a method used for methamphetamine analysis of wipes using liquid-liquid extraction.	GC-MS Make not specified	Colorado, USA	Van Dyke et al. 2008 [16]
Clothing	Cotton sweatshirt spiked with known concentrations of methamphetamine. Fabric and clothing (including jeans, t-shirts, undergarments, socks, and car seat covers) from suspected or known methamphetamine users or cooks.	Liquid extraction of swatches using different solvents.	GC-MS Agilent Technologies 6890N GC 5975 with Agilent Mass Selective Detector	Alabama, USA	Keasey 2011 [46]
Clothing	Cotton denim (tight weave), cotton blanket (looser weave), fire department turnout jackets, law enforcement ballistic vest, Nomex coveralls and polyester/cotton coveralls contaminated with laboratory-generated methamphetamine aerosol.	Liquid extraction of swatches using NIOSH method 9111, a method used for methamphetamine analysis of wipe samples.	LC-MS Make not specified	Colorado, USA	Serrano et al. 2012 [15]
Textile	Loosely woven upholstery fabric (19% cotton, 79% olefin, 2% rayon). Transfer of methamphetamine from fabric to skin.	Not applicable	Radioactivity using a PerkinElmer Tri-Carb 2900 TR liquid scintillation spectrometer	California, USA	Salocks et al. 2014 [47]
Textile	Low-pile, synthetic carpet contaminated with methamphetamine from simulated “smoking”. Cotton glove used to determine the dermal transfer of methamphetamine from a contaminated surface.	Liquid extraction of swatches and surface wipe sampling media using NIOSH method 9111.	LC-MS Make not specified	Colorado, USA	Van Dyke, Martyny, and Serrano 2014 [17]
Clothing/Textile	Polyester baby blankets, polyester baby toy ‘book’, polyester woman’s shirts, cotton baby T-shirt and cotton/polyester blend upholstery which were exposed to methamphetamine gas.	Liquid extraction of swatches with 6.5 mL ethyl acetate with 10 µL of 1000 ppm internal standard bromofluorobenzene (BFB) solution in ethyl acetate.	GC-MS Agilent, model not specified	Missouri, USA	Morrison, Shakila, and Parker 2015 [27]
Textile	Carpet and rugs from contaminated property.	Liquid extraction of surface wipe samples or bulk samples using NIOSH method 9111.	LC-MS Agilent 1290 Infinity UHPLC system with 6460/6470 Triple Quadrupole LC-MS electrospray source	Australia	Wright et al. 2019 [18]
Textile	Soft toys from contaminated property.	Personal sampling pump (1 L/min flow rate) with ORBO™-49P (XAD^®^-2 resin) sampling tubes followed extraction using NIOSH method 9111.	LC-MS Agilent 1290 Infinity UHPLC system with 6460/6470 Triple Quadrupole LC-MS electrospray source	Australia	Wright et al. 2021 [13]

Note: DESI-MS = desorption electrospray ionisation mass spectrometry, GC-MS = gas chromatography-mass spectrometry, HPLC-FL = high-performance liquid chromatography with fluorescence detection, HPLC-UV = high-performance liquid chromatography with ultraviolet detection, LC-MS = liquid chromatography-mass spectrometry, NIOSH = National Institute for Occupational Safety and Health, UHPLC = ultra-high performance liquid chromatography.
